# Hidden localization motifs: naturally occurring peroxisomal targeting signals in non-peroxisomal proteins

**DOI:** 10.1186/gb-2004-5-12-r97

**Published:** 2004-11-30

**Authors:** Georg Neuberger, Markus Kunze, Frank Eisenhaber, Johannes Berger, Andreas Hartig, Cecile Brocard

**Affiliations:** 1 Research Institute of Molecular Pathology (IMP), Dr Bohr-Gasse 7, A-1030 Vienna, Austria; 2 Brain Research Institute, Department of Neuroimmunology, Medical University Vienna, Spitalgasse 4, A-1090 Vienna, Austria; 3 Max F Perutz Laboratories, Institute of Biochemistry and Molecular Cell Biology, University of Vienna and Ludwig-Boltzmann-Forschungsstelle für Biochemie, Dr Bohr-Gasse 9, A-1030 Vienna, Austria

## Abstract

**Background:**

Can sequence segments coding for subcellular targeting or for posttranslational modifications occur in proteins that are not substrates in either of these processes? Although considerable effort has been invested in achieving low false-positive prediction rates, even accurate sequence-analysis tools for the recognition of these motifs generate a small but noticeable number of protein hits that lack the appropriate biological context but cannot be rationalized as false positives.

**Results:**

We show that the carboxyl termini of a set of definitely non-peroxisomal proteins with predicted peroxisomal targeting signals interact with the peroxisomal matrix protein receptor peroxin 5 (PEX5) in a yeast two-hybrid test. Moreover, we show that examples of these proteins - chicken lysozyme, human tyrosinase and the yeast mitochondrial ribosomal protein L2 (encoded by *MRP7*) - are imported into peroxisomes *in vivo *if their original sorting signals are disguised. We also show that even prokaryotic proteins can contain peroxisomal targeting sequences.

**Conclusions:**

Thus, functional localization signals can evolve in unrelated protein sequences as a result of neutral mutations, and subcellular targeting is hierarchically organized, with signal accessibility playing a decisive role. The occurrence of silent functional motifs in unrelated proteins is important for the development of sequence-based function prediction tools and the interpretation of their results. Silent functional signals have the potential to acquire importance in future evolutionary scenarios and in pathological conditions.

## Abstract 2

Functional but silent peroxisomal targeting signals have been found in non- peroxisomal proteins. This discovery has important implications for sequence-based signal prediction and for evolution.

## Background

For an increasing number of otherwise uncharacterized protein sequences from genome-sequencing projects, function assignment is attempted solely with *in silico *prediction methods, as reliable and cost-effective large-scale experimental methods are not available. In addition to sequence homology and annotation transfer considerations [1], these function assignments increasingly rely on algorithms that recognize protein-sequence features responsible for posttranslational modifications, subcellular localization and interactions with specific domains of other proteins.

Although considerable effort has been invested in achieving low false-positive prediction rates, our experience with tools for recognizing glycosyl phosphatidylinositol (GPI) lipid [2,3] and myristoyl [4-6] anchor attachment sites and for predicting potential targets for PTS1-dependent translocation to peroxisomes [7] shows that a small but noticeable number of proteins without appropriate biological context (for example with contradictory subcellular localization or in taxa without the modifying enzyme or receptor) are systematically hit by these tools. For example, we found more than a dozen metazoan lysozymes [7,8], known extracellular proteins, that are predicted to have carboxyl termini with a functional peroxisomal targeting signal 1 (PTS1) region.

Are these false-positive predictions? All three of the sequence-analysis tools mentioned above check query sequences for a recognition pattern that is explicitly described in terms of its physical properties and it is possible to check the concordance between pattern descriptions and query sequence individually. Nevertheless, this visual inspection is frequently unable to rationalize the findings as false-positive predictions, as all known components of the pattern appear to be present. Even in the case of high accuracy of the prediction tool, an erroneous prediction cannot be excluded. Alternatively, these predicted sequence motifs may occur by chance and be functional in an appropriate test system, but still have no biological meaning because the necessary cellular context is absent *in vivo*. Only experimental tests can resolve this contradiction. As a case study, we report the results of an experimental analysis that demonstrates the existence of naturally occurring peroxisomal targeting signals in several known non-peroxisomal proteins. We also discuss the evolutionary perspective of functional localization signals in unrelated proteins as well as the consequences for experimental localization determination and function prediction from sequence.

The major mechanism for targeting proteins to the matrix of peroxisomes, which are membrane-bounded organelles [9] of eukaryotic cells, is initiated in the cytoplasm by interaction of the receptor protein peroxin 5 (PEX5) with the carboxy-terminal signal PTS1 on the target protein [10,11]. This signal consists of three regions of sequence comprising approximately 12 residues [12,13]. It is composed of the most carboxy-terminal tripeptide (classically, the -SKL terminus), preceded by a region of around four residues (which interact with the surface at the mouth of the PEX5 binding cavity), and a solvent-accessible (or easily unfoldable) stretch of around five residues further upstream. The PTS1-prediction program 'PTS1' [14] identifies PTS1 signals in query protein sequences by evaluating their carboxy-terminal ends with respect to features necessary for interaction with the tetratricopeptide repeats of PEX5. The predictor's scoring function searching for this motif within the 12 carboxy-terminal residues achieves an estimated sensitivity of 90% and a selectivity above 99% [7].

## Results

### The carboxyl termini of several non-peroxisomal proteins interact with PEX5

Screening of SWISS-PROT [15] entries with the PTS1 predictor identified proteins from several families that are clearly not peroxisomal but score highly and are predicted as PEX5 targets [7,8]. We were not able to rationalize these results as false predictions as the proteins' carboxyl termini did not deviate from the generalized PTS1 sequence pattern [13]. To verify whether these proteins could indeed interact with PEX5, we tested the carboxyl termini of seven representative proteins in a yeast two-hybrid system: hen egg-white lysozyme (P00698, secreted); dog lysozyme C from milk (P81708); tyrosinase from human (P14679, a melanosomal type I membrane protein); frog tyrosinase (Q04604); *Drosophila *sevenless (P13368, a large transmembrane protein required for photoreceptor development); precursor of lysosomal bovine cathepsin D (P80209); and a mitochondrial ribosomal protein from yeast (P12687). We also examined the carboxyl terminus of a mouse dihydrofolate reductase construct with an added SKL peptide, which has been shown not to be imported into yeast peroxisomes [16,17].

Depending on their taxonomic origin, the carboxyl termini of the eukaryotic sequences were assayed for interaction with the tetratricopeptide repeat domains of either human or yeast PEX5 using published methodologies [12]. The query sequences, along with prediction scores and measured β-galactosidase activities, are summarized in Table 1. The results show that all peptide sequences interact with the PTS1-receptor PEX5 in the two-hybrid system. Hence, the carboxy-terminal sequences of these assayed non-peroxisomal proteins fulfill the requirements to function as PTS1 signals.

### The accessibility of the PTS1-like carboxyl terminus is critical

The fact that the peroxisomal translocation machinery fails to import naturally occurring mature proteins carrying PTS1 signals into peroxisomes *in vivo *could be explained by the non-accessibility of their carboxyl termini. These could either be hidden in the native structure of the mature protein or of its functional complexes, or competing translocation machineries could lead to a removal of the respective proteins from the cytosol before their recognition by PEX5.

The first possibility is exemplified by DHFR-SKL. The carboxy-terminal 16 residues of the DHFR-SKL construct (EKGIKYKFEVYEK**SKL**, sequences appended to DHFR are in bold type, see results in Table 1) interact with yeast PEX5 in the two-hybrid test but *in vivo *the complete construct is not imported into peroxisomes, thus confirming the prediction [16,17]. For comparison, it should be noted that two other DHFR-derived constructs with slightly longer carboxyl termini (IKYKFEVYEK**GGKSKL **and IKYKFEVYEK**KNIESKL**) are predicted to be peroxisomally targeted. Their scores calculated with the PTS1 predictor [7] are 13.2 and 9.9, respectively (compare with data in Table 1). They were experimentally shown [17] to be translocated to peroxisomes. In the native three-dimensional structure of DHFR [18], the carboxyl terminus is part of a β-sheet that is buried in the fold, deprived of flexibility and accessibility. Seemingly, this structure prevents the carboxy-terminal appended residues SKL in the construct from entering the PEX5 binding cavity, whereas slightly longer carboxyl termini may do. In our two-hybrid test system, the carboxy-terminal 16-mers are always considered exposed as, in the non-native sequence environment of the carboxyl terminus of the GAL4 activation domain, they are free from interfering or blocking structural features. Thus, DHFR-SKL fails to be imported into peroxisomes because its carboxyl terminus is sequestered in the structure of the mature protein.

### Competing targeting signals prevent translocation into peroxisomes despite the presence of PTS1-like carboxyl termini

Alternatively, functional PTS1 signals can be overruled by other localization signals [7]. For instance, distribution of the mammalian alanine-glyoxylate amino transferase (AGT) between peroxisomes and mitochondria is regulated by the variable occurrence of an amino-terminal mitochondrial targeting signal in the mature protein (depending on the usage of two alternative transcription initiation sites) [19,20].

Does a naturally occurring PTS1-like carboxyl terminus of a clearly non-peroxisomal protein that is capable of interacting with PEX5 indeed lead to *in vivo *import of the respective protein, provided that a potentially overruling sequence signal is eliminated? A set of three target proteins with amino-terminal leader sequences was chosen from Table 1. Chicken lysozyme (SWISS-PROT id P00698), a secreted enzyme, is one of the best characterized proteins and has an apparently accessible carboxyl terminus as deduced from its three-dimensional structure (Protein Data Bank (PDB) number 1H6M [21]). The corresponding carboxy-terminal 16-mer produces moderate β-galactosidase activity in the yeast two-hybrid assay (most of the other proteins in Table 1 appear to interact even more strongly with PEX5). Human tyrosinase (P14679) is a melanosomal marker protein that functions in the formation of pigments such as melanins. Yeast 60S ribosomal protein L2 (P12687), or MRP7, is a component of the large subunit of the mitochondrial ribosome.

**Figure 1:**
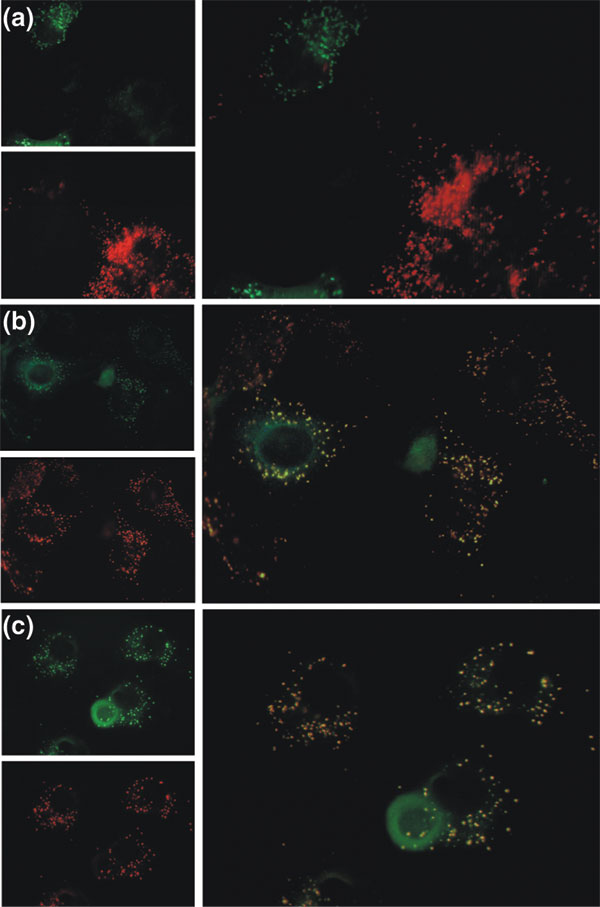
Targeting of GFP-tyrosinase and GFP-lysozyme to peroxisomes in human cells. Fluorescence of human COS7 cells expressing **(a) **GFP-lysozyme or DsRed2-SKL; **(b) **GFP-tyrosinase and DsRed2-SKL; or **(c) **GFP-lysozyme and DsRed2-SKL. Cells were observed 36 h after transfection (magnification 60 ×). Separate small images of the GFP fluorescence (green) and DsRed2 fluorescence (red) are shown to the left of each main picture, in which the two fluorescent images are overlaid. Areas in which red and green fluorescence coincide show as yellow. (a) Control experiments reveal that expression of GFP-lysozyme is an adjunct to the cellular punctuate fluorescence pattern independently of the presence of DsRed2-SKL. The figures show a punctate fluorescence pattern for GFP fusions with (b) human tyrosinase and (c) chicken lysozyme. Both proteins co-localize with DsRed2-SKL in human peroxisomes as demonstrated by the fluorescence overlay. Owing to the evolutionary conservation of PEX5 within the metazoans [7,13,33], a chicken protein (lysozyme) can be assayed in a human cell line and the species barrier is not an issue in this study.

**Figure 2:**
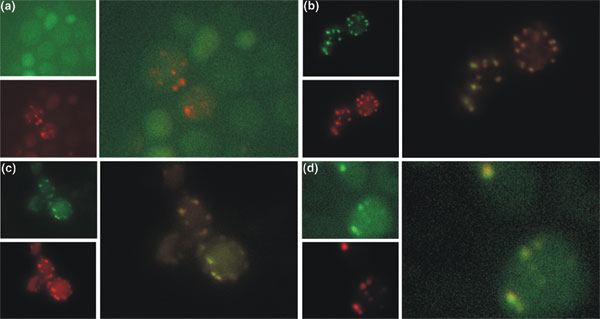
Targeting of GFP-MRP7 and GFP-GSA to peroxisomes in yeast cells. Fluorescence of CB80 yeast cells expressing **(a) **GFP and DsRed-SKL; **(b) **GFP-SKL and DsRed-SKL; **(c) **GFP-MRP7 and DsRed-SKL; or **(d) **GFP-GSA and DsRed-SKL. Transformed cells were cultured on oleate and observed live for fluorescence. Control experiments (a) show that GFP co-localizes with Ds-Red-SKL only when the sequence -SKL is appended at its extreme carboxyl terminus (b). The figures reveal a punctuate fluorescence pattern for GFP fused to the yeast mitochondrial ribosomal protein L2 encoded by *MRP7 *(c) or to the bacterial enzyme glutamate-1-semialdehyde 2,1-aminomutase (GSA) (d). Both fusion proteins co-localize with DsRed-SKL in yeast peroxisomes. GFP fused to GSA without its carboxy-terminal -AKL gave rise to a diffuse (cytosolic) fluorescence pattern (data not shown).

Green fluorescent protein (GFP) was appended to the amino terminus of each of the selected proteins. It can be assumed that translocation into the endoplasmic reticulum (ER) or mitochondria is disrupted by the resulting shift of the signal peptide from the amino terminus to the center of the protein. The resulting molecules are expected to be redirected into peroxisomes if their carboxyl termini can act as PTS1 signals. Targeting of the GFP-constructs *in vivo *was indeed confirmed by co-localization with a peroxisomal DsRed2-SKL construct in COS7 cells for the metazoan enzymes (Figure 1) and with DsRed-SKL in yeast cells for the *Saccharomyces cerevisiae *protein (Figure 2). Thus, the PTS1 signals at the carboxyl termini of the assayed proteins are normally suppressed by alternative amino-terminal targeting sequences. A similar mechanism can be inferred for other eukaryotic SWISS-PROT proteins listed in Table 1, although steric carboxy-terminal accessibility or other factors might also play a role.

### Functional PTS1 sequences can occur in organisms without peroxisomes

The occurrence of silent PTS1s without a targeting role raises the question of whether such signals can also evolve in organisms that do not carry peroxisomes. To test this hypothesis, we extended Table 1 with a set of four predicted carboxyl termini from prokaryotic enzymes: *Escherichia coli *glutamate-1-semialdehyde 2,1-aminomutase (P23893), *E. coli *transaldolase A (P78258), *Methanopyrus kandleri *riboflavin synthase (NCBI-Refseq accession NP_613646) and *Archaeoglobus fulgidus *2-nitropropane dioxygenase (NCBI-Refseq accession NP_070998). Indeed, these proteins harbor carboxyl termini that qualify as PTS1 signals (lower part of table 1). As confirmation, for the bacterial protein glutamate-1-semialdehyde 2,1-aminomutase (GSA) we used the same methodology for subcellular localization determination as for yeast MRP7. The resulting GFP-GSA construct is also imported into peroxisomes (Figure 2), demonstrating that its PTS1-like carboxyl terminus is functional in the mature protein.

## Discussion

In families of orthologous proteins, peroxisomal location and its targeting signal in the amino-acid sequence are not necessarily conserved. For example, in plants the five enzymes of the glyoxylate cycle are localized to peroxisomes, but in *S. cerevisiae *three of the five (aconitase, isocitrate lyase, and the respective malate dehydrogenase isoform) could not be found in peroxisomes [22]. Thus, it is not surprising to find sporadically occurring PTS1 signals in protein families (see some examples in Table 1).

In dually localized proteins such as AGT [23], the PTS1 signal has a biological role as a targeting signal. However, the carboxyl termini of the proteins from Table 1 do not seem to fulfill any specific targeting function. We suggest that these PTS1 signals occur as a result of neutral mutation. The presence of a functional PTS1 signal would not lead to evolutionary pressure in this context because mislocalization is prevented by overriding the function of these sequences either by alternative exposure of amino-terminal signals or by steric carboxy-terminal inaccessibility.

**Figure 3:**
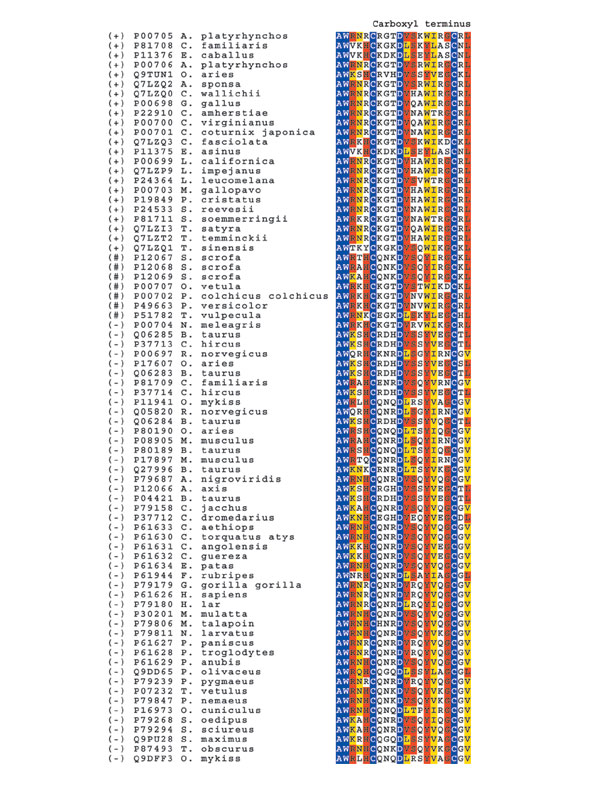
Multiple alignment of lysozyme carboxyl termini. A screen of the SWISS-PROT database [15] for proteins that harbour PTS1 signals produced a set of lyosozymes, well characterized secreted enzymes that are not usually found in peroxisomes. Rather than occurring sporadically, a large fraction of the known sequences from this family was obtained using the PTS1 prediction tool [7]. Moreover, these hits could not be rationalized as false positives as they did not deviate from the PTS1 sequence motif [11-13]. The multiple alignment shows intact vertebrate lysozyme carboxy-terminal 20-mers (with accession number and species name) retrieved from the SWISS-PROT database. From a total of 76 entries, 23 have predicted PTS1s (score > 0; at the top, marked with '+'), seven are in the twilight zone (-10 < score < 0; in the middle, marked with '#') and 46 are not predicted (score < -10; at the bottom, marked with '-'). There appears to be an overlap between the PTS1 motif and sequence variability within the lysozyme family. For example, the absolutely conserved cysteine near the carboxyl terminus is needed for the formation of a disulfide bridge in the mature protein [21]. This cysteine also meets the requirement for a small residue at the antepenultimate position of the PTS1 sequence.

The case of lysozyme is particularly noteworthy because a large number of homologous proteins were systematically hit when performing a SWISS-PROT screen using the prediction tool (30 cases with putative PTS1s and 46 other lysozyme carboxyl termini are shown in Figure 3). Because of the close relationship of the originating species and the occurrence of several isozymes, the lysozyme sequences in the multiple alignment share a high degree of similarity. The PTS1 carboxyl termini seem to be a mimicry of the sequence needed to support structural features of the protein. The cysteine at the antepenultimate position, which is present as part of a disulfide bridge [21] in the final secreted form of lysozyme, happens to fulfill the need for a small residue at the respective PTS1 location. The PTS1 is mostly functional, with a positively charged or amidic penultimate amino acid and the correct hydrophobic carboxy-terminal residue, which is the case for a large proportion of the lysozymes. Note that the disulfide bridge will not be formed in our GFP-lysozyme test case because translocation of the fusion protein into the endoplasmic reticulum is prevented.

We conclude that a PEX5-interacting sequence can evolve simply by mutational alterations in the carboxy-terminal region of a protein. Although shuffling of a carboxy-terminal exon cannot be excluded for other examples, the fact that the open reading frames (ORFs) of the carboxy-terminal exons for human tyrosinase (GenBank accession AP000720.4), fly sevenless (GenBank accession AE003484.2) and chicken lysozyme (GenBank accession AF410481.1) reach far into the functional domains of their proteins, rather supports an evolutionary mechanism of several point substitutions. The occurrence of functional PTS1 sequences in non-eukaryotic species further supports a stochastic model for the evolution of PEX5-interacting protein carboxyl termini.

In non-globular regions of proteins, sequences that code for targeting to other subcellular compartments, or for posttranslational modifications, might appear in similar ways during evolution. For example, the sequence motif coding for amino-terminal *N*-myristoylation of glycines behaves as an exchangeable functional module, as protein families do exist where it has been substituted by alternative sequence determinants that facilitate membrane association [6]. This is exemplified by the *Arabidopsis thaliana *Rab5 ortholog Ara7 and its paralog Ara6. Ara7 is geranylgeranylated on carboxy-terminal cysteines just as Rab5 is in other species. However, the closely related paralog Ara6 lacks the carboxy-terminal cysteines and has an experimentally verified amino-terminal myristoylation motif [24].

Many of these signals seem to remain silent under normal physiological conditions (as is the case for the PTS1 signal in some metazoan lysozymes) but have the potential to become important in some future evolutionary scenarios or in pathological situations. Alternatively, the PTS1 signal might have become obsolete and the corresponding sequence segment is now subject to evolutionary alterations. Apparently, the cell exploits only a fraction of the potential molecular capabilities of its proteins.

Futhermore, subcellular targeting is organized in a hierarchy of cellular recognition mechanisms. The co-translational sorting into the ER serves as a first decision node. Posttranslational processes such as interaction with chaperones, folding, and covalent modifications are concomitant with the appropriate exposure of targeting signals. The amino-terminal signals are made first and are therefore favored when it comes to recognition by receptors. PEX5 needs only to categorize the remaining unsorted proteins with accessible carboxyl termini into 'stay here' or 'let's go into peroxisomes'. This might also explain why the PTS1 signal is comparatively short and permissive for a wide range of residues.

Clearly, the fact that functional sequences for subcellular targeting occur in unrelated proteins needs to be considered for prediction-tool development. The construction of a negative learning set (sequences without the specific localization signal) on the basis of proteins with differing cellular localization is problematic. For example, a set of non-peroxisomal but organellar localized [25], viral [26] or bacterial sequences might contain a considerable number of proteins that potentially interact with PEX5. Thus, such a set does not directly qualify for automated learning procedures or the assessment of false-positive prediction [27,28].

Surprisingly, when Maurer-Stroh and Eisenhaber applied their myristoylation site predictor for eukaryotic proteins to bacterial proteomes [5], systematic hits were found despite the absence of known amino-terminal *N*-myristoyltransferases (NMT) in bacteria. Are these false-positive predictions? A literature search revealed that myristoylation by host NMTs has physiological relevance for several secreted proteins of intracellular bacterial parasites [5]. Thus, the sequence motif coding for amino-terminal *N*-myristoylation is typical for eukaryotes but occurs also in bacteria. In many cases, it remains without phenotypic effect for bacteria but may become evolutionarily important in the case of host-parasite interactions.

In the case of the endothelin-converting enzyme 1 and the neprilysin-like zinc metallopeptidase family, the carboxy-terminal CXAW motif is a valid prenylation motif. This carboxy-terminus is functionally hidden because the protein is exported to the extracellular side of the cytomembrane and the carboxy-terminal residues are apparently involved in folding and enzyme function [29].

Clearly, the accessibility of the recognition motif in the substrate protein to the respective receptor or protein-modifying enzyme is a major issue. For PTS1 signal prediction from the amino-acid sequence, carboxy-terminal exposure needs to be assessed both from the steric point of view as well as in the context of competing translocation mechanisms. Analyzing only the carboxy-terminal dodecamer peptide [7,13] might not suffice for reliable prediction of accessibility to the receptor, but a full solution would require sufficiently accurate three-dimensional structure prediction.

In databases, it should also be routine to flag proteins that contain several competing targeting signals with differing priority. Finally, silent localization signals might become active in mutant protein constructs and lead to non-native localizations, an issue that needs to be assessed especially in localization screens of proteins with uniformly incorporated fluorescent dyes such as GFP. It cannot be excluded that the subcellular location of a considerable number of proteins has not been correctly determined in published large-scale studies that rely on this methodology [30,31].

To conclude, sequence segments coding for subcellular targeting or for posttranslational modifications can occur in proteins that are not substrates in either of these processes. Accurate prediction techniques reveal candidate proteins carrying hidden sequence signals. Many of these can be experimentally confirmed. In the case of the PTS1 predictor program, there is no reasonable argument to assume a difference in prediction accuracies for real and hidden PTS1s as, in both cases, productive interaction of the carboxyl terminus with PEX5 is the criterion for a functional PTS1.

## Materials and methods

### Cloning procedures

Oligonucleotides were purchased from MWG Biotech (Munich, Germany). The *E. coli *strain DH5α, Bethesda Research Laboratories) was used for all transformations and plasmid isolations. For the yeast two-hybrid-assay, the hybridized oligonucleotide pairs coded for the carboxy-terminal 16-mers of the selected proteins flanked by *Bam*HI (5') and *Eco*RI (3') restriction sites. Each oligonucleotide pair was introduced into a *Bam*HI-*Eco*RI-digested pGAD.GH fragment, generating plasmids containing the Gal4p activation domain in addition to the desired carboxy-terminal 16-mer extension (Gal4pAD-16mer). All pGAD.GH constructs were sequenced (VBC Genomics, Vienna, Austria). The plasmids pAH987 and hP87 contain the binding domain of Gal4p fused to the TPR domain of *S. cerevisiae *or *Homo sapiens *PEX5, respectively (Gal4pBD-TPR) [12].

Chicken cDNA for the amplification of lysozyme was generated from chicken oviduct using Tripure (Invitrogen) according to the manufacturer's instructions. Reverse transcription was performed using RNA-PCR Core Kit (Applied Biosystems) following the manufacturer's instructions. For the amplification of tyrosinase, we used cDNA from the melanoma cell line 29 WUBI (generous gift of Walter Berger, Vienna). The coding regions of lysozyme and tyrosinase were gained by PCR (for oligonucleotide primers see Table 2) using the Advantage cDNA Polymerase Mix kit from Clontech and the GeneAmp PCR-system from Perkin Elmer. The PCR-fragments were cloned into the pCR2.1 vector (Invitrogen) by T/A cloning and sequenced as control (VBC Genomics). The fragments containing the lysozyme or tyrosinase coding regions were excised with *Eco*RI/*Bam*HI and ligated into pEGFP-C1 (Clontech). The DsRed2-SKL construct was obtained by PCR using Pfu-polymerase (Promega) and the plasmid pDsRed2-C1 (Clontech) as template (for oligonucleotides, see Table 2). The PCR fragment and the plasmid were both cut with *Eco*47-3/*Xho*I and the PCR fragment encoding the carboxy-terminal SKL was introduced to replace the original DsRed2 end sequence. The final plasmid encodes the DsRed2-SKL protein under the control of the cytomegalovirus promoter.

Standard procedures were used for cloning of the GFP-MRP7 and GFP-GSA constructs including control sequencing (VBC Genomics). The plasmids expressing GFP and GFP-SKL under control of the *MLS1 *promoter were described previously [32]. The DNA fragment coding for DsRed-SKL was obtained by PCR (for oligonucleotides, see Table 2; template pDsRed, Clontech) and cloned (*Bam*HI-and partially with *Pst*I) after the *MLS1 *promoter in the vector YEplac181. DNA fragments coding for MRP7 and GSA were obtained by PCR (see Table 2 for oligonucleotide sequences) and cloned (*Bam*HI-*Sph*I) in-frame with GFP to give rise to the expression of GFP-MRP7 and GFP-GSA, respectively, all of them under the control of the *MLS1 *promoter.

### Yeast two-hybrid assay

According to the Matchmaker two-hybrid protocol, yeast strain PCY3 (*MAT*α, *his3*Δ200, *ade2*-101, *trp1*Δ63, *leu2*, *gal4*Δ, *gal80*Δ, *lys2*::*GAL1-HIS3*, *ura3*::*GAL1-lacZ*) [12] was transformed with the Gal4pAD-16mer constructs (plasmid pGAD.GH) together with either pAH987 or hP87. Yeast transformants were selected and grown on minimal medium containing 2% glucose and supplemented with bases and amino acids as required (SC-leu-trp). For quantitative measurement of β-galactosidase activity in accordance with published techniques [12], yeast cells were grown in selective medium (SC-leu-trp) overnight at 30°C, diluted to *A*_600 _= 0.3 into the same medium and finally harvested at absorptions of *A*_600 _between 0.9 and 1.1.

### *In vivo *localization study in COS7 cells

COS7 cells were transfected with the pEGFP-C1-constructs and DsRed2-SKL by electroporation using 920 μF and 220 mV (Gene pulser II, Bio-Rad), grown on coverslips for 36 h, washed, fixed with 0.5% formaldehyde in PBS for 15 min and covered with geltol. Cells were analyzed using the Olympus BX51 fluorescence microscope (60 × enlargement).

### *In vivo *localization study in yeast cells

The yeast strain used in this study is *S. cerevisiae CB80 *(*MATa*, *ura3-52*, *leu2-1*, *trp1-63*, *his3-200*). Yeast transformants were selected and grown on minimum medium containing 0.67% yeast nitrogen bases without amino acids (Difco Laboratories), 2% glucose and amino acids (20-150 μg/ml) as required (SC-leu-ura). For fluorescence microscopy, yeast cells were grown at 30°C with shaking in selective media with 0.5% glucose as sole carbon source until the glucose concentration was very low (0.05%, usually 16 h), harvested by centrifugation and resuspended in the original volume of induction medium containing 0.67% yeast nitrogen bases without amino acids, 0.1% yeast extract, 30 mM potassium phosphate pH 6.0, 0.125% oleate, 0.2% Tween-80 and amino acids as required. Cells were grown for 16 h in induction medium and observed live for fluorescence. Briefly, cells were collected by centrifugation and washed twice in water. Cell pellets were resuspended in induction medium without oleate and aliquots were spotted onto multitest slides (ICN Biochemicals) previously coated with concanavalin A (6 mg/ml, Sigma). Cells were allowed to attach for 5 min at room temperature and the slides were washed twice with induction medium and a coverslip applied for observation. Fluorescence was viewed with a Zeiss Axioplan 2 fluorescence microscope using a 63 × (1.4 NA) lens. Digital images were captured with a Quantix CCD camera using Lightview software without further modification. The pictures were mounted and false-color overlays were made in Adobe Photoshop.

## Figures and Tables

**Table 1:** Results of the yeast-two hybrid interaction assays with PEX5

		Yeast PEX5			Human PEX5				
				
Species	Accession	Score*	Activity^† ^(Units/mg protein)	Standard deviation	Score*	Activity^† ^(Units/mg protein)	Standard deviation	Carboxyl terminus	Description
*Canis familiaris*	P81708	-	-	-	0.17	25	2	HCKGKDLSKYLASCNL	Lysozyme
*Drosophila melanogaster*	P13368	-	-	-	6.70	29	11	PLKDKQLYANEGVSRL	Sevenless protein
*Gallus gallus*	P00698	-	-	-	2.02	73	4	RCKGTDVQAWIRGCRL	Lysozyme
*Rana nigromaculata*	Q04604	-	-	-	0.13	91	15	LLMEAEDYQATYQSNL	Tyrosinase
*Homo sapiens*	P14679	-	-	-	4.01	242	10	LLMEKEDYHSLYQSHL	Tyrosinase
*Bos taurus*	P80209	-	-	-	7.04	310	58	FDRDQNRVGLAEAARL	Cathepsin D
*Saccharomyces cerevisiae*	P12687	2.72	482	37	-	-	-	KVEVIARSRRAFLSKL	Mitochondrial ribosomal protein L2, or MRP7
Synthetic construct	DHFR-SKL	11.51	195	45	-	-	-	EKGIKYKFEVYEKSKL	DHFR-SKL
*Escherichia coli*	P23893	4.81	270	26	11.35	473	57	DINNTIDAARRVFAKL	Glutamate-1-semialdehyde 2,1-aminomutase
*E. coli*	P78258	-9.46	164	31	5.59	566	70	FAVDQRKLEDLLAAKL	Transaldolase A
*Methanopyrus kandleri*	NP_613646	6.08	45	8	10.41	358	46	GMGRREGHPDVGPARL	Riboflavin synthase
*Archaeoglobus fulgidus*	NP_070998	7.57	206	19	-1.36	0	NA	EEVIRKIAEGLNKAKF	2-nitropropane dioxygenase

All eukaryotic target sequences (characterized by species, SWISS-PROT or NCBI-Refseq accession number, score from the PTS1 predictor [7], carboxy-terminal sequence and description) were tested for interaction with the tetratricopeptide (TPR) repeat domain of human PEX5, except for P12687 and DHFR-SKL where the corresponding TPR domains were derived from yeast PEX5. The prokaryotic proteins were assayed using PEX5 from both yeast and human. As the estimated length of the PTS1 signal is 12 carboxy-terminal residues [13], we chose the carboxy-terminal 16-mers to be sure that we have included the complete motif-carrying segment. *A PTS1 prediction score above zero is considered predictive of a functional PTS1 signal; a score between -10 and 0 is considered a 'twilight zone' prediction. It should be noted that the negative score for the DHFR-SKL carboxyl terminus in its context is generated by the PTS1 predictor [7] solely by terms that evaluate its potential accessibility for PEX5. ^†^A yeast-two hybrid assay is considered positive if the measured β-galactosidase activity is clearly greater than zero. Experience from previous test series suggests a lower limit of around 10 Miller Units per mg protein [12] for the detection of a productive interaction. The measured β-galactosidase activities (including standard deviations) range from weak (P81708, P13368) to strong (P80209, P12687).

**Table 2:** Oligonucleotides used for the amplification of the GFP-constructs

Construct	Forward primer	Reverse primer
EGFP-tyrosinase	GAATTCAATGCTCCTGGCTGTTTTGTACTG	GGATCCTTATAAATGGCTCTGATACAAGCTG
EGFP-lysozyme	GAATTCCATGAGGTCTTTGCTAATCTTGGT	GGATCCGGCAGCTCCTCACAGCCG
GFP-MRP7	CGGGATCCAATGTGGAATCCTATTTTACTAGATAC	GGGCATGCTCAAAGCTTGCTCAAAAAAGCCCG
GFP-GSA	CGGGATCCAATGAGGAAGTCTGAAAATCTTTACCAG	GGGCATGCTCACAACTTCGCAAACACCCGACG
DsRed2-SKL (COS7 cells)	CGGCTAGCGCTACCGGTCGCCACCATGGCC	CGTCTCGAGTTATAATTTGGACAGGTGGTGGCGGCC
DsRed-SKL (yeast cells)	AGATCTATGGTGAGGTCTTCCAAG	CTGCAGTTATAATTTGGATAGGATCCCAAGGAACAGATGGTGGCG
